# An Improved Routing Schema with Special Clustering Using PSO Algorithm for Heterogeneous Wireless Sensor Network

**DOI:** 10.3390/s19030671

**Published:** 2019-02-07

**Authors:** Jin Wang, Yu Gao, Wei Liu, Arun Kumar Sangaiah, Hye-Jin Kim

**Affiliations:** 1 Hunan Provincial Key Laboratory of Intelligent Processing of Big Data on Transportation, School of Computer & Communication Engineering, Changsha University of Science & Technology, Changsha 410000, China; jinwang@csust.edu.cn; 2 College of Information Engineering, Yangzhou University, Yangzhou 225000, China; gaoyuyz@163.com (Y.G.); yzliuwei@126.com (W.L.); 3 School of Information Science and Engineering, Fujian University of Technology, Fuzhou 350000, China; 4 School of Computing Science and Engineering, Vellore Institute of Technology (VIT), Vellore 632014, India; sarunkumar@vit.ac.in; 5 Business Administration Research Institute, Sungshin W. University, Seoul 100744, Korea

**Keywords:** WSN, energy efficiency, energy center, PSO, mobile sink, network lifetime

## Abstract

Energy efficiency and energy balancing are crucial research issues as per routing protocol designing for self-organized wireless sensor networks (WSNs). Many literatures used the clustering algorithm to achieve energy efficiency and energy balancing, however, there are usually energy holes near the cluster heads (CHs) because of the heavy burden of forwarding. As the clustering problem in lossy WSNs is proved to be a NP-hard problem, many metaheuristic algorithms are utilized to solve the problem. In this paper, a special clustering method called Energy Centers Searching using Particle Swarm Optimization (EC-PSO) is presented to avoid these energy holes and search energy centers for CHs selection. During the first period, the CHs are elected using geometric method. After the energy of the network is heterogeneous, EC-PSO is adopted for clustering. Energy centers are searched using an improved PSO algorithm and nodes close to the energy center are elected as CHs. Additionally, a protection mechanism is also used to prevent low energy nodes from being the forwarder and a mobile data collector is introduced to gather the data. We conduct numerous simulations to illustrate that our presented EC-PSO outperforms than some similar works in terms of network lifetime enhancement and energy utilization ratio.

## 1. Introduction

In recent years, the increased performance of the microminiature sensor in terms of memory capability and sensitivity and the decreased price have caused the widespread use of the self-organized wireless sensor networks (WSNs) [1,2,3,4]. They commonly consist of plenty of sensors monitoring the area of interest (AoI) and use special routing protocols to realize the information exchange. The sensors are usually randomly deployed in tough environment using aircraft and organize networks by themselves. Due to the advantages of WSNs such as convenient deployment, self-organization, and low price, they have been employed in forest fire detection [5,6], environment monitoring [7,8], medical systems and healthcare [9,10,11], and smart homes [12,13,14].

Because of the harsh environment of the deployed sensors in some applications, it is impossible for people to change the batteries of sensors. Therefore, energy efficiency is an important factor for us to design routing protocols. Much researches have been focused in energy conservation and many routing schemas have been proposed. Two-tier [15,16,17,18,19,20,21] routing schema usually divides the sensors into two types, member nodes and CHs by clustering. In a two-tier WSN, member nodes transmit their sensor data to the corresponding CH by single or multiple hop communication, and the CHs take the responsibility to process the data fusion and forward the data to the sink. The benefit of clustering can be summarized as follows: (1) Data fusion can be conducted in CHs to fuse the data from their members. Therefore, redundant data can be filtered and the transmission burden of CHs is relieved. (2) It simplifies the topology of the network and reduce the control messages because local nodes only need to know its corresponding CH. Meanwhile, it enhances the scalability of the network. (3) Communication bandwidth is conserved because the intracluster communication usually uses a time division multiple access (TDMA) schema.

However, in the two-tier schema, nodes close to the CHs will consume more energy and cause energy holes because they relay the data for the outer nodes [15,16]. Unbalanced energy consumption may cause premature death of nodes and cut down the network’s lifetime. In order to achieve the goal of energy balancing, one valid method is to utilize the mobile sink for energy balancing [18,22,23,24,25,26]. As the energy efficient routing problem in WSNs has been proved to be a NP-hard problem [27], some biologically inspired algorithms [28,29,30,31,32,33,34] are also introduced to solve it. Some work combines ant-colony algorithm and aims to find an optimal path for data transmission to save and balance the energy [28,29]. In this paper, we propose an improved routing schema utilizing energy center-based clustering to achieve the goal of energy efficient and energy balance. The protocol was conducted by rounds and during the first several rounds, CHs were elected in accordance with the location of nodes using geometric partitioning. We calculated the optimal communication distance for multi-hop communication. Then we used the PSO algorithm to search the energy centers of the network and chose the nodes closest to the energy centers as the CHs. A low energy protection mechanism was also used to avoid weak nodes becoming relay nodes. Extensive simulations were conducted and the result proved that our presented protocol owns a better performance in several metrics such as lifetime and energy consumption of the network. We could summarize our contribution as follows:

- We explored the optimal communication distance for multi-hop transmission.
- PSO algorithm was utilized for the energy centers searching to select the CHs.
- A protection mechanism is proposed to avoid low energy nodes becoming relay nodes.
- Plenty of simulations were performed and we compared them with some similar work such as Azharuddin et al. [32], Variable Dimension based Particle Swarm Optimization (VD-PSO) [34].

## 2. Related Work

### 2.1. Routing Protocols Based on Heuristic Algorithm

Low Energy Adaptive Clustering Hierarch (LEACH) [15] is a classic routing algorithm and it firstly introduced the idea of clustering to achieve energy efficient. In LEACH, CHs are random selected and the node chose the closest CH as its destination by single hope communication. LEACH is much more energy efficient than some flat routing protocols such as flooding, grossing, SPIN, and DD because it avoids the direct communication between sensors and the sink. However, the method of random CHs selection brings about the uneven distribution of CHs and it is not suitable for large scale WSNs.

Fuzzy Logic based Hexagonal Geographical Adaptive Fidelity (FGAF-HEX) [35] is a routing method which utilizes the fuzzy logic system and geography information to achieve the target of energy efficient and energy balance. The main contributions of the work can be summarized as follows. It divides the whole sensor field into several regular hexagons in accordance with communication range and arbitrary nodes in adjacent hexagons could intercommunicate. In this way, each hexagon could only maintain one active node and keep other nodes sleeping to alleviate data redundancy and conserve energy. Fuzzy logic system is utilized to balance the energy consumption in each hexagon field. Experiment results illustrate that the presented method outperforms some traditional algorithms such as Geographical Adaptive Fidelity (GAF), Diagonal Geographical Adaptive Fidelity (DGAF), Hexagonal Geographical Adaptive Fidelity (GAF-HEX).

In [36], a straight-line routing using random-walk is proposed. The main idea of this protocol is that the event and query paths are straight and they are likely to interest in a plane. In this protocol, a candidate area is ensured according to the inside and outside bands which are determined by the neighbor distance. The node with the farthest neighbor distance and biggest residual energy in the candidate area is select as the next hop. Simulation results demonstrate that it outperforms some classic random-walk routing protocols such as rumor routing (RR) in aspects of network lifetime.

Mobile sink could further balance the network’s energy consumption. In [22], a data gathering schema called Energy-Aware Path Construction (EAPC) is proposed using mobile sink for energy efficient. The schema is composed of three parts to calculate a moving path for the mobile sink. During the initial phase, a minimal spanning tree is constructed to connect all the sensor nodes. Then a set of collection points (CPs) are elected according to the benefit index during the CP selection phase and the ordinary tree is decomposed into many subtrees. Finally, a convex polygon is constructed based on the CPs. The mobile sink moves along the convex polygon to access each CP and gathers the data in each round. Simulation result proves that the presented schema owns an enhanced performance in aspects of lifetime.

A hierarchical routing algorithm which based on cluster-chain is proposed in [23], the algorithm contains three steps to construct the topology of the network. During the clustering and chain formation phase, each node calculates its cluster head selection value (CHSV) in accordance with the residual energy, neighbor distance, and the amount of data generation. The node with maximal CHSV will be elected as the cluster head. Then the greedy algorithm is introduced to form chains for intracluster communication. Finally, a mobile agent (MA) is used to collect data from CHs along a dynamic path which is determined by signal strength, residual energy level and path loss. Simulation results describe that they proposed algorithm outperforms some similar work such as LEACH, Energy Efficient Cluster-chain based Protocol (ECCP), and Power-Efficient Gathering in Sensor Information Systems (PEGASIS).

A dynamic route adjustment method is proposed in [24], the algorithm divides the nodes into several groups according to the location information and chose the node with most residual energy as the CH among each group. A greedy algorithm is used for CHs to generate a chain to transmit data to the mobile sink and the CH which is nearest to the mobile sink is the chain leader. The topology of the network will not change until the chain leader changes.

### 2.2. Routing Protocols Based on Metaheuristic Algorithm

Ant Colony Optimization (ACO) which is inspired by ants foraging has been adopted by several literatures for routing path selection. In [28], the authors proposed an algorithm combined the fuzzy logical system with ACO. The fuzzy logical system is used for the CHs selection according to the input features such as distance to BS, residual energy, and node degree. The ACO is used for optimal routing path discovery for each node by using the probability function. In [31], the authors use the type-2 fuzzy logical system combined with ACO to further improve the network’s performance, and the Sugeno fuzzy complement operator is used to update the pheromone concentration. In [30], the author defines the CHs routing problem in WSN as the traveling salesman problem (TSP) and ACO is utilized to search for an optimal trajectory with shortest distance.

Particle swarm optimization (PSO) is another metaheuristic algorithm widely applied among many optimization problems. In [31], Kuila et al. proposed an energy efficient routing schema. Their work’s contribution is to establish the mapping relation between particles and sensors. The network is constructed by two different types of sensors, ordinary nodes and gateways, and each particle represents the whole solution for the routing path. The dimension of the particle is the number of gateways and each dimension represents the index of the candidate relay node. The fitness function mainly contains two parts, Maxdis and Maxhop, and the ultimate goal is to minimize the fitness function. In [32], the authors proposed an energy efficient and energy balanced algorithm based on Kuila et al. and they pay more attention to the energy balance. In [33], a novel clustering method is created by using region partition line represented by a tuple $L=(x,y,\theta_{x},\theta_{y})$. PSO algorithm is used to adjust the region partition line in order to achieve better network performance. In [34], an algorithm called VD-PSO is presented by Wei Wang et al. In VD-PSO, the dimension of each particle is double the number of rendezvous points and it stores the coordinate of CHs. Due to the uncertainty of the number of the rendezvous points, the dimension of particles is different, therefore, the authors proposed a special way to update the speed and location of the particles. In [37], the author proposed a coverage hole patching scheme using PSO to search coverage holes and fix it with a mobile agent.

### 2.3. Routing Protocols for Energy Holes Avoiding

Energy holes in WSNs are generally caused by the unbalanced energy consumption of nodes and they may greatly shorten the lifetime of the network if they are not well addressed. Many works aim to solve the problem of energy holes. In [38], the authors propose a method to preserve nodes near energy holes by avoiding the topology reformation overhead. In [39], the authors adopt a special method to divide the whole network into many concentric circular tracks and sectors which decrease the energy consumption via filtering redundant data. In [40], the authors introduce random walk (RW) into network energy balancing. Meanwhile, the average communication distance between nodes and the average communication distance between RWs and sink are optimized to reduce the energy consumption. In [41,42], sink mobility technology is adopted to address the energy holes problem. Additionally, the visualization for multidimension data [43] is also helpful to display the simulation results.

## 3. System Model

### 3.1. Network Model

A heterogeneous square area is considered as the network model of our presented schema, and plenty of sensors are deployed using a random way. We initially set the sink at the center of the sensor field. In every round, each node needs to transmit its sensor data to the sink by single or multi-hop transmission. A sensor can choose a CH within its communication range otherwise it will transmit the data to a relay node for forwarding. CHs are usually nodes with high energy and distribute evenly by the PSO algorithm mentioned in Section 4, and they take the responsibility for data fusing and transmit the fused data to the sink. CHs apply the TDMA mechanism to control their member nodes’ transmission time, and once a node joins a cluster, the corresponding CH will allocate a TDMA time slot to it. With the purpose of facilitating the experiments, we make the following assumptions:

- Sensors have the knowledge of their own location according to an equipped GPS and they know their neighbors’ location during the initial phase by information exchange.
- The sink has the geography information of all the sensor and in every round, each sensor will report its residual energy to sink in transmitted data.
- We assume that the network is in a favorable transmission environment and don’t consider the collision during the transmission. The radio channel is symmetric [44].
- The clocks of sensors are synchronized using a GPS module or a time synchronization method such as Flooding Time Synchronization Protocol (FTSP) or Glossy [45].

### 3.2. Energy Model

We adopt the same radio model to calculate the energy consumption which is mentioned in [46,47]. The energy of transmitter $E_{Tx}$ can be calculated by two different equations in accordance with their communication distance. Once the signal is generated by the transmitter, the amplifier will strengthen it using different power according to the transmission distance. Therefore, we adopt two different models for transmission. The free space model is adopted if the distance is less than a threshold value $d_{0}$, otherwise we adopt multi-path fading model to calculate the energy consumption. The energy used for l bit data transmission through a distance d is given by follows:

(1) $$E_{Tx}(l,d)=\left\{ \begin{array}{ll} l\cdot E_{elec}+l\cdot \varepsilon_{fs}\cdot d^{2} & if,d<d_{0}\\ l\cdot E_{elec}+l\cdot \varepsilon_{mp}\cdot d^{4} & if,d\geq d_{0} \end{array}\right.$$

where $E_{elec}$ denotes the power the transmitter and receiver use. $\varepsilon_{fs}$ and $\varepsilon_{mp}$ denote two different power the amplifier uses.

The threshold value $d_{0}$ can be calculated using the following formula:

(2) $$d_{0}=\sqrt{\frac{\varepsilon_{fs}}{\varepsilon_{mp}}}$$

Then the energy receiver uses to receive *l* bit data is calculated by

(3) $$E_{Rx}(l)=l\cdot E_{elec}$$

## 4. Our Proposed EC-PSO Algorithm

### 4.1. Overview of Traditional PSO Algorithm

**Figure 1:**
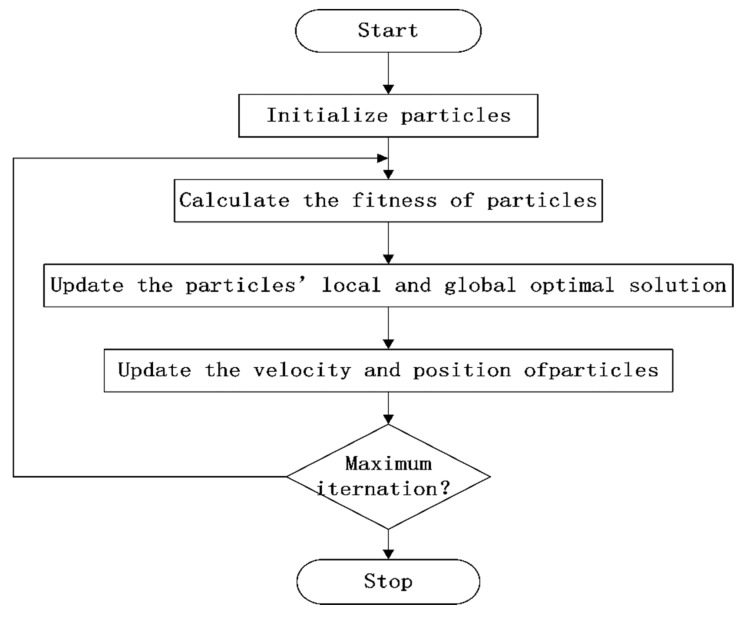
Particle swarm optimization (PSO) workflow.

PSO is an intelligent heuristic algorithm which uses the wisdom of swarm. In PSO, the solution of the problem is represented by the particles and the fitness function is defined to evaluate the performance of the solution. The local and global optimal solutions are chosen to update the location of the particles during each iteration. After a number of iterations, the particles will find the optimal solution in the searching space. The workflow of PSO is illustrated as Figure 1.

### 4.2. Clustering with Non-Linear Programming

Our main goal for clustering is to maximize the energy of the nodes which are close to the CHs. Therefore, the energy holes could be avoided to become the traffic hubs. We use a Boolean variable $b_{ij}$ to represents whether node i is close to $CH_{j}$.

(4) $$b_{ij}=\left\{ \begin{array}{ll} 1 & \mathrm{If}\;\mathrm{node}\;i\;\mathrm{is}\;\mathrm{close}\;\mathrm{to}\;CH_{j},\\ & \forall i,j:1\leq i\leq N,1\leq j\leq M\\ 0 & \;\mathrm{Otherwise} \end{array}\right.$$

where *N* is the number of nodes and *M* is the number of CHs. Let *Aveg_Energy* be the average energy of nodes close to the CHs and it can be calculated as:

(5) $$Aveg\_Energy=\frac{\sum_{i=1}^{M}\sum_{j=1}^{N}E_{i}\cdot b_{ij}}{\sum b}$$

where $E_{i}$ denotes the residual energy of node i.

Therefore, the non-linear programming (NLP) for the clustering can be formulized as:

(6) Maximize(Aveg_Energy)

### 4.3. Basic Phases

**Figure 2:**
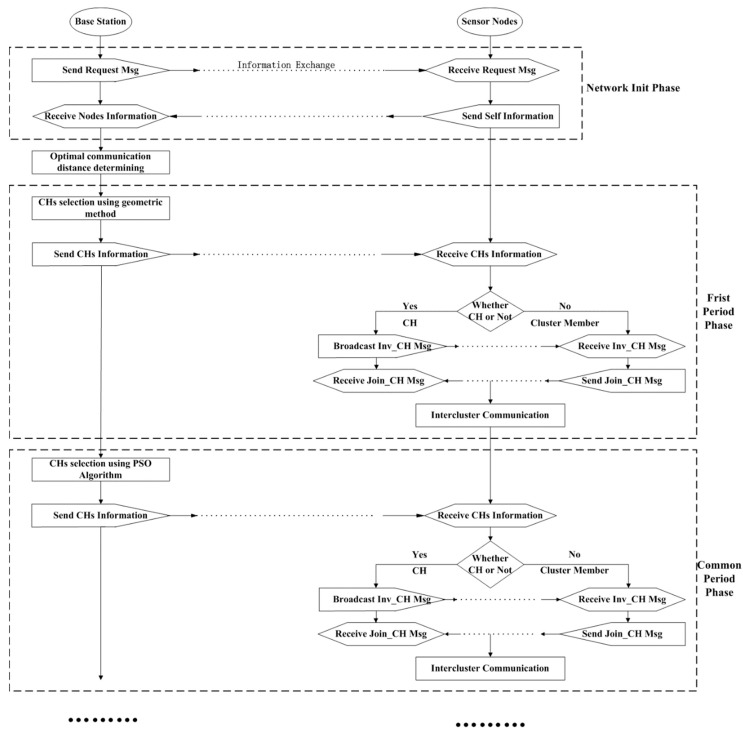
Network workflow.

During the initial phase when all the nodes are deployed, sensors exchange their own information with their neighbors. Since the initial energy of sensors are equal, the sink makes the first period CHs selection by geometrical method to evenly distribute the CHs. We use grid to divide the field and nodes close to the intersection point of the grid are chosen as CHs. The first period includes about 50 rounds and after that the energy of network is heterogeneous. The round number 50 is determined by simulations and 50 is a suitable round number which ensure that the energy of different regions of the network achieve significant difference. After that the CHs selection will adopt PSO algorithm to choose nodes close to the energy centers as CHs. Ordinary nodes choose the closest CH to join using optimal distance communication. Afterwards, a greedy algorithm is used for CHs to form a chain or tree to transmit data to the sink instead of long-distance communication. With the purpose of conserving energy, the network topology maintains for several rounds to avoid frequent control messages broadcasting, and the process is shown in Figure 2.

### 4.4. Optimal Communication Distance Determining

Sensors transmit data to the CHs using single or multiple hops communication, and the average hop distance is a crucial factor to the energy consumption. If the average hop distance is small, more relay nodes are needed, and much energy is consumed in forwarding. However, if the average hop distance is large, it may cause long-distance communication and consume much energy. We assume that the distance between source node and the target is M and the average hop distance is m. The overall energy consumption for one-bit data transmission can be given by:

(7) $$E_{total}=\left\{ \begin{array}{ll} \frac{M}{m}[E_{elec}+E_{fs}m^{2}]+(\frac{M}{m}-1)E_{elec} & if,m<d_{0}\\ \frac{M}{m}[E_{elec}+E_{mp}m^{4}]+(\frac{M}{m}-1)E_{elec} & if,m\geq d_{0} \end{array}\right.$$

**Figure 3:**
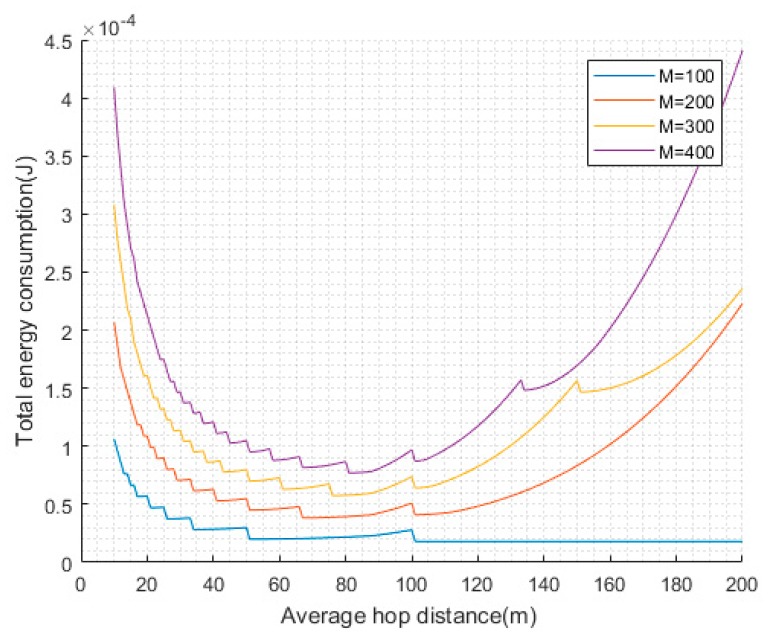
Optimal communication distance.

We can get the relationship between average hop distance and total energy consumption according to Figure 3. From Figure 3, we can clearly see that when the communication distance is about 90, the energy can be used more efficiently.

### 4.5. First Period CH Selection

**Figure 4:**
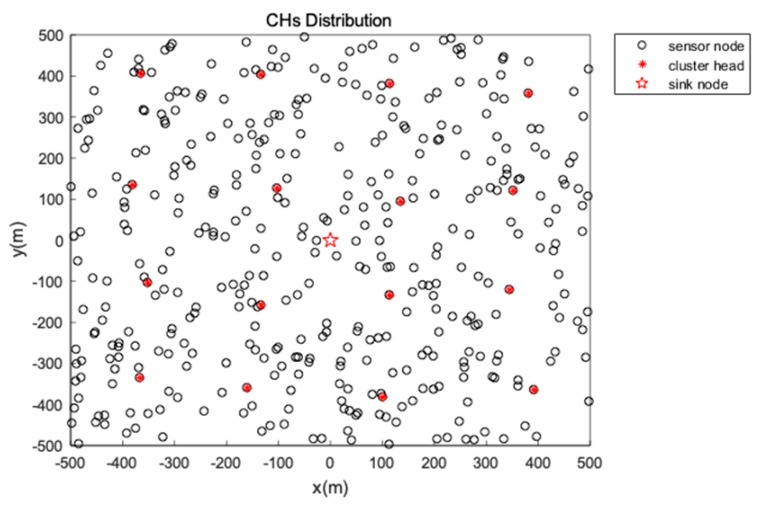
First period CHs distribution.

During the initial phase after the sensors are deployed, the energy of the network is homogeneous and no energy centers exist. Therefore, we use geometric method to distribute the CHs evenly and the number of the CHs is given using the Formula (8).

(8) $$CH\_N=\left\lfloor \sqrt{N\cdot Per\_CH}\right\rfloor$$

where Per_CH denotes the percentage of CHs in sensors and N represents the number of sensors. The distribution of CHs after first period CHs selection is shown as Figure 4.

**Figure 5:**
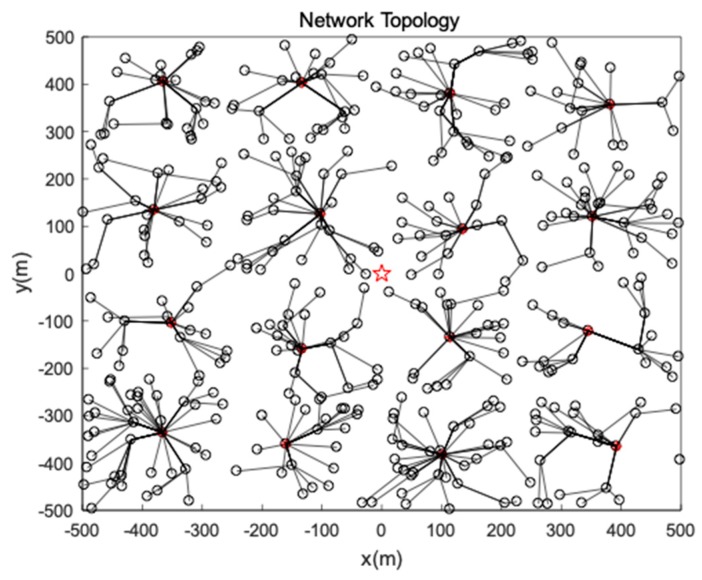
Network topology.

After CHs are elected, each CH broads a Cluster_Join message and nodes join a closest CH in accordance with the receiving signal strength. The optimal communication distance is used by each node to forward data to the CH and the topology is described in Figure 4 and Figure 5. Then the greedy algorithm is used to generate a chain or tree for the CHs to transmit data to sink as is discussed in Section 4.7. The data fusion is conducted in each CH in order to preserve energy.

**Figure 6:**
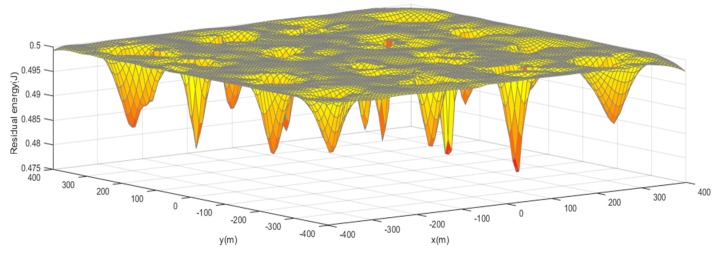
Energy distribution.

When the network spends its first period, it enters into a steady phase. Then the PSO algorithm is executed for clustering and the topology of the network will maintain for 20 rounds. We visualize the residual energy of the network using a 3D picture as is shown in Figure 6. It illustrates that there are energy holes near the CHs because nodes close to CHs take the role of forwarder and they consume more energy. Our main purpose is to select the CHs near the energy centers to avoid energy holes and balance the energy consumption of nodes.

### 4.6. Energy Center Based CHs Selection using PSO

In order to better illustrate our proposed EC-PSO, we firstly introduce some parameters which will be used in Table 1.

Many literatures use the location weight as the center, however the location of sensors is almost stationary and the location centers cannot react the distribution of energy. In this section, PSO algorithm is utilized to search the energy centers for CHs selection. Each particle denotes a whole solution for the energy centers and the dimension of the particle is double the number of CHs. Our algorithm uses a swarm of M particles and it is shown as Formula (9).

(9) $$S=\left[\begin{array}{c} P_{1}\\ P_{2}\\ \vdots \\ P_{M} \end{array}\right]=\left[\begin{array}{cccc} P_{1,1} & P_{1,2} & \cdots & P_{1,N}\\ P_{2,1} & P_{2,2} & \cdots & P_{2,N}\\ \vdots & \vdots & & \vdots \\ P_{M,1} & P_{M,2} & \cdots & P_{M,N} \end{array}\right]$$

where M is the number of particles and N is the number of CHs, and $P_{i,j}$ represents a 2-tuple that contains the ordinate of each energy center and is given as Formula (10).

(10) $$P_{i,j}=(X_{center\_j},Y_{center\_j})$$

The object of the PSO algorithm is to find the energy centers and we expect the global energy of the energy center is as high as possible, so we define the fitness function as follows.

(11) $$F=\sum_{1}^{M}\sum_{s\in C_{j}}E_{residual}\frac{1}{N_{C_{j}}}$$

where $C_{j}$ is a collection which contains nodes about two hops away from $P_{i,j}$. We use the following steps to conduct the PSO algorithm.

**Step 1**: In order to achieve evenly distributed CHs, we set the initial location of the particles in regular positions and the velocity of each particle is chosen in a random way. Every round, each node reports its status which contains the information of its ID, residual etc.

**Step 2**: After the sink receives the message, the value of fitness function is calculated using the Formula (11). Particle $P_{i}$ compares the value with its individual optimal solution and choose the better one as its $P_{ibest}$. In the same way, it compares the value with the global optimal solution to choose the best particle as the $G_{best}$.

**Step 3**: The velocity of the particles can be updated using Formula (12) and their positions can be updated using Formula (13).

(12) $$V_{i}(t+1)=\omega V_{i}(t)+c_{1}\times rand()\times (P_{ibest}-P_{i}(t))+c_{2}\times rand()\times (G_{best}-P_{i}(t))$$

(13) $$P_{i}(t+1)=P_{i}(t)+V_{i}(t+1)$$

where ω represents the self-adapting parameter and $c_{1}$, $c_{2}$ are two positive constants.

**Step 4**: When each particle gets a new value, it will check the distance of its 2-tuples $P_{i,j}$ and $P_{i,k}$. If the distance between the 2-tuples is less than a threshold value, we reinitialize one of the 2-tuples in order to get the evenly distributed energy centers as much as possible.

**Step 5**: Then, it goes to step 2 and terminate until it reaches the maximal iteration number. We select the nodes closest to the energy centers as the CHs, and the sink broadcasts the result of CHs selection.

A few low energy nodes are inevitably mixed in energy centers, so we take a mechanism to protect low energy nodes. We set a threshold using the following formula and keep nodes whose energy is lower than threshold from forwarding.

(14) $$E_{th}=\frac{\sum_{1}^{N}E_{residual}}{N}$$

### 4.7. Intercluster Communication

Traditional data collection schema usually uses static sink for information gathering and it easily causes hot spots problem and shorten the network’s lifetime. In this paper, a mobile data collector is utilized to gather the information from CHs according to a certain rule. At the beginning of the first round, the mobile data collector is placed at the center of the network area.

Intercluster routing is conducted using the following steps.

**Step 1**: The collector always moves to the energy center with highest average energy and the average energy of the energy center can be calculated using Formula 15.

(15) $$Avg_{i}=\sum_{s\in C_{i}}E_{residual}\frac{1}{N_{C_{i}}}$$

**Step 2**: The mobile agent broadcasts the information of all the CHs and its own location. Once the CHs receives the broadcast information they will wake up and other nodes still keep sleep to save energy.

**Step 3**: Each CH chooses a close CH as the forwarder, and the forwarder needs to be more close to the mobile data collector compared with the source node.

**Step 4**: The CH whose closest neighbor is the sink takes the charge of transmitting the data to the sink.

**Figure 7:**
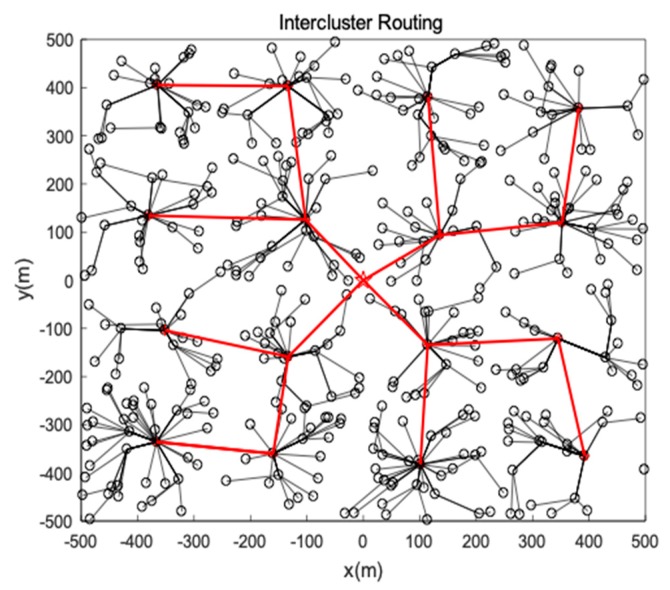
Intercluster routing.

The network topology after intercluster routing is shown as Figure 7.

## 5. Performance Evaluation

To evaluate our proposed EC-PSO algorithm, we compare it with some other works, namely VD-PSO and the algorithm Azharuddin et al. proposed.

The experiment environment is Matlab simulator and 400 sensors are randomly placed in a $1000\times {1000\;m}^{2}$ field. We set the initial energy of each sensor 0.5 J and some relevant parameters are described from Table 2.

**Figure 8:**
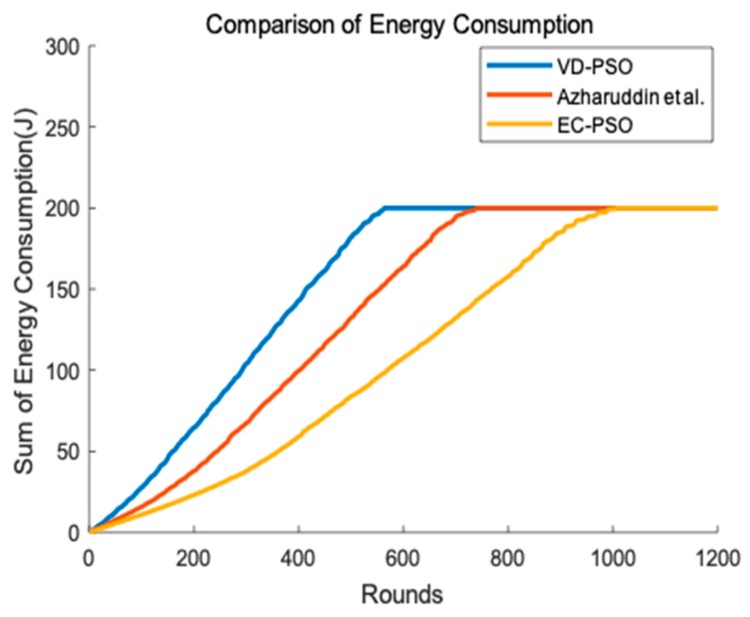
Energy consumption of the network.

Figure 8 describes that with the number of rounds increasing, the total energy of the network increases simultaneously in different algorithms. VD-PSO only adopts rendezvous points for mobile agent to gather information and nodes far away from the rendezvous points will spend much energy for long-distance data transmission. Therefore, it consumes the most energy. Both the algorithm Azharuddin et al. proposed and EC-PSO introduce the clustering method to reduce the energy consumption and simplify the topology of the network. However, the algorithm Azharuddin et al. proposed doesn’t consider the distribution of CHs and a few nodes still need to transmit their data to the forwarder by long distance communication. EC-PSO consumes less energy compare to the other two algorithms because it uses optimal distance communication to transmit data to the CHs with less hops and much energy are conserved from unnecessary forwarding. The CHs EC-PSO elects are also more reasonable than the other two algorithms.

**Figure 9:**
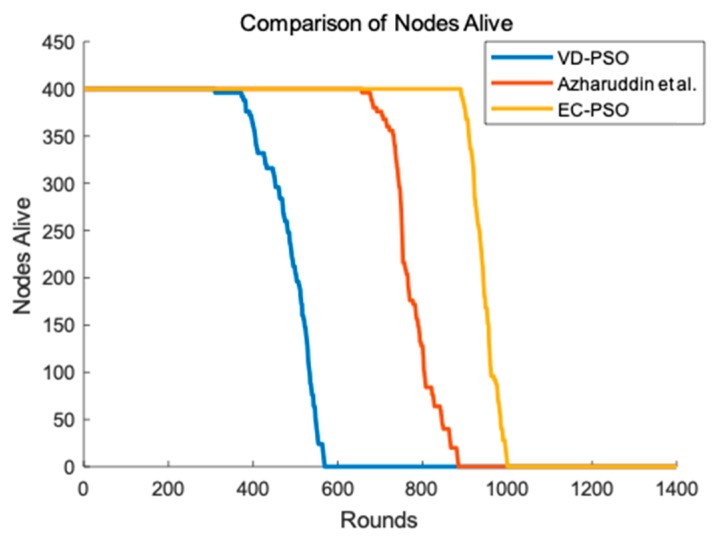
Network lifetime. VD-PSO: Variable Dimension based Particle Swarm Optimization; EC-PSO: Energy Centers Searching using Particle Swarm Optimization.

Lifetime is a crucial standard to evaluate the performance of network and it is usually defined as the time when the first node dies. We can clearly see from Figure 9 that our proposed EC-PSO has a better performance in terms of network lifetime. The first node dies around about 400 rounds for VD-PSO, and about 700 rounds for the algorithm Azharuddin et al. proposed. However, in EC-PSO, it can be about 900 rounds when the first node dies. Both VD-PSO and the algorithm Azharuddin et al. proposed do not consider the energy balance and they both consume more energy than EC-PSO. Additionally, EC-PSO uses protection mechanism to protect weak nodes and energy holes are avoided when transmitting. Therefore, EC-PSO achieves the obvious improvement in enhancing the lifetime of the network.

**Figure 10:**
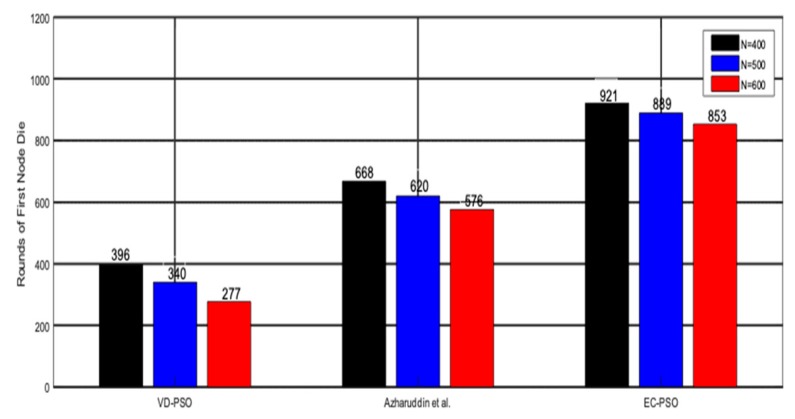
Rounds of first node die.

First node dies (FND) is also an important evaluation criterion for network performance in WSNs. In Figure 10, we test different algorithms under the circumstances of different number of sensor nodes. Simulation result shows that when the number of sensor nodes rises, our algorithm still has a better performance than the other two algorithms because we take the energy balance into consideration.

**Figure 11:**
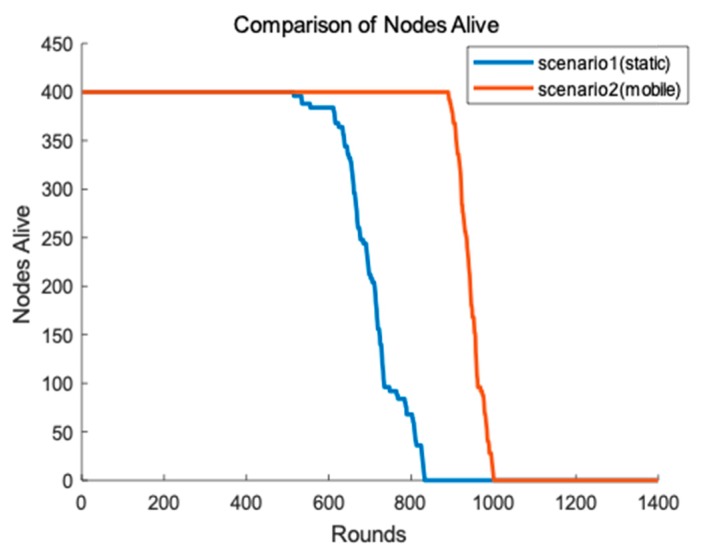
Lifetime of two different scenarios.

In order to demonstrate that the mobile data collector has improved the performance of the network, we conduct two different scenarios. One of the scenarios is that the sink is fixed at the center of the network and CHs transmit data to the sink using greedy algorithm. One CH chooses a closest CH towards the sink node as a relay node until the data is transmit to the sink. Another scenario is that we have mentioned in Section 4.7. Result of the simulation is shown as Figure 11. In Figure 11, we can clearly see that the round of first node die in scenario 1 is much earlier than that of scenario 2 because the static sink causes the hot spots problem and make nodes close to the sink premature death. However, the mobile data collector may result in packages loss in transmission because of its movement and unpredictability. Different application can make a choice according to the requirement of network lifetime and stability.

**Figure 12:**
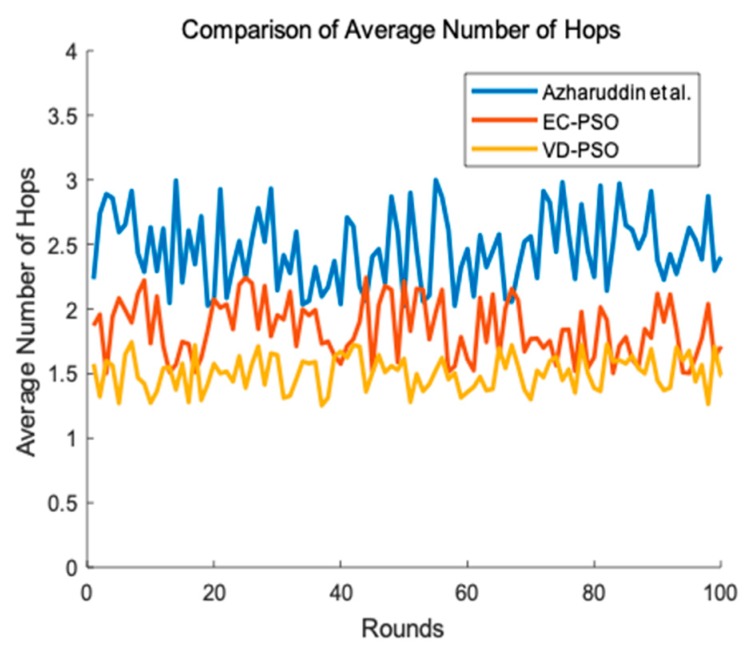
Average number of hops between different algorithm.

Additionally, we explore the total traffic and end-to-end delay of the network. We firstly defined the average number of hops as the average number of the packages which are forwarded. Bigger the average number of hops is, busier the traffic of the network is. Meanwhile, there is a positive correlation between the average number of hops and end-to-end delay. In Figure 12, We can clearly see that VD-PSO owns the best performance in terms of average number of hops because it sets rendezvous points for mobile data collector and most of the sensors using single hop to send data to the sink when the sink accesses the rendezvous point. Our proposed EC-PSO adopts multi-hop communication to transmit data to the sink and the CHs need to forward their members’ packages. Therefore, average number of hops in EC-PSO is bigger than that in VD-PSO. The algorithm Azharuddin et al. proposed also uses multi-hop communication, however their communication distance is not scheduled. Therefore, its average number of hops is biggest in the three algorithms.

## 6. Conclusions

In this paper, we have proposed an energy center-based routing protocol for WSN. The network experiences two periods and two different strategy for clustering are adopted. During the first period, we used the geometric method to elect the CHs and the topology maintains for several rounds. After the energy of the network became heterogeneous, the special clustering using PSO was executed to search energy centers for CHs election. Common clustering routing protocols are likely to cause energy holes and EC-PSO avoids these energy holes. Additionally, random reinitialization were used to avoid CHs getting too close and a protection mechanism using threshold value was utilized to keep the low energy nodes from forwarding. A mobile data collector which is attracted by the energy center with highest average energy was adopted to gather the sensor data. Through numerous simulations, we can conclude that our proposed EC-PSO has a better performance in terms of energy consumption and network lifetime.

## Figures and Tables

**Table 1:** PSO Parameters.

Parameter	Definition	Value
*S*	The particles which represent the solution	Random generation
*M*	The number of particles	50
*N*	The number of CHs	16
$P_{i,j}$	The ordinate of energy center	
$C_{j}$	Neighbors of energy center *P*	
*V*	The velocity of particles.	Random generation
$P_{ibest}$	The optimal local solution	
$G_{best}$	The optimal global solution	
ω	The inertia factor	0.5
$c_{1}$	The weight factor of $P_{ibest}$	0.4
$c_{2}$	The weight factor of $G_{best}$	0.6

**Table 2:** Simulation parameters.

Parameter Name	Value
Network size (R)	1000 × 1000 m^2^
Number of nodes (N)	400
Initial energy (*E*_0_)	0.5 J
Energy consumption on circuit (*E_elec_*)	50 nJ/bit
Free-space channel parameter ($\varepsilon_{fs}$)	10 pJ/bit/m^2^
Multi-path channel parameter ($\varepsilon_{mp}$)	0.0013 pJ/bit/m^4^
Packet length (*l*)	1000 bits
Distance threshold (*d*_0_)	$\sqrt{\frac{\varepsilon_{fs}}{\varepsilon_{mp}}}$ m
